# The role of Patient Health Engagement Model (PHE-model) in affecting patient activation and medication adherence: A structural equation model

**DOI:** 10.1371/journal.pone.0179865

**Published:** 2017-06-27

**Authors:** Guendalina Graffigna, Serena Barello, Andrea Bonanomi

**Affiliations:** 1 Department of Psychology, Università Cattolica del Sacro Cuore, Milan, Italy; 2 Department of Statistical Sciences, Università Cattolica del Sacro Cuore, Milan, Italy; IRCCS Istituto Auxologico Italiano, ITALY

## Abstract

**Background:**

Increasing bodies of scientific research today examines the factors and interventions affecting patients’ ability to self-manage and adhere to treatment. Patient activation is considered the most reliable indicator of patients’ ability to manage health autonomously. Only a few studies have tried to assess the role of psychosocial factors in promoting patient activation. A more systematic modeling of the psychosocial factors explaining the variance of patient activation is needed.

**Objective:**

To test the hypothesized effect of patient activation on medication adherence; to test the the hypothesized effects of positive emotions and of the quality of the patient/doctor relationship on patient activation; and to test the hypothesized mediating effect of Patient Health Engagement (PHE-model) in this pathway.

**Material and methods:**

This cross-sectional study involved 352 Italian-speaking adult chronic patients. The survey included measures of i) patient activation (*Patient Activation Measure 13 –short form*); ii) Patient Health Engagement model (*Patient Health Engagement Scale*); iii) patient adherence (*4 item-Morinsky Medication Adherence Scale*); iv) the quality of the patients’ emotional feelings (*Manikin Self Assessment Scale*); v) the quality of the patient/doctor relationship (*Health Care Climate Questionnaire*). Structural equation modeling was used to test the hypotheses proposed.

**Results:**

According to the theoretical model we hypothesized, research results confirmed that patients’ activation significantly affects their reported medication adherence. Moreover, psychosocial factors, such as the patients’ quality of the emotional feelings and the quality of the patient/doctor relationship were demonstrated to be factors affecting the level of patient activation. Finally, the mediation effect of the Patient Health Engagement model was confirmed by the analysis.

**Conclusions:**

Consistently with the results of previous studies, these findings demonstrate that the Patient Health Engagement Model is a critical factor in enhancing the quality of care. The Patient Health Engagement Model might acts as a mechanism to increase patient activation and adherence.

## Introduction

An increasing body of scientific research today examines the factors and interventions affecting patients’ ability to self-manage and adhere to treatment.[1,2] Patient activation is considered to be the most reliable indicator of the willingness and ability to manage health and care autonomously.[3–5] The patient activation theory has been developed by Hibbard and Mahoney.[2] It describes an incremental process, which patients undergo when becoming protagonists of their care management. Specifically, this theory is rooted in the concepts of self-efficacy [6,7] and locus of control, [8,9] and in the transtheoretical model of change.[10] It refers to “the individual’s knowledge, skill and confidence in managing his/her own health and care”[11]. Drawing on this theory, Hibbard and Mahoney [2] developed the Patient Activation Measure *(PAM-13)*, which is a 13-item Likert self-reported questionnaire. The PAM-13 is widely used to measure the level of empowerment and self-management of chronic care patients. [5] The scale features four levels of patient activation describing different levels of patients’ readiness to assume an active role in their care management.

Previous studies have shown the role of patients’ activation in influencing their adherence to treatment prescriptions. [12,13] Moreover, studies have found that patients actively involved in their care plans are also more likely to trust their clinicians [14] and less likely to experience adverse clinical events and hospital readmissions.[15] Furthermore, making patients active in their healthcare is also recognized as a key strategy with which to make healthcare more sustainable by reducing healthcare-related costs.[16] Numerous studies have demonstrated empirically that patients more activated in their care are also more likely to enact preventive behavior such as having regular check-ups, screenings, and immunizations.[17–20] More highly activated people are also significantly more likely to enact healthy behaviors such as following a healthy diet and taking regular exercise. [21] Moreover, those who are more activated are more likely to avoid health-damaging behaviors like smoking and substance abuse.[22] In general, studies have demonstrated the association of a high level of patient activation with positive clinical outcomes in several populations across chronic conditions [23–27]. This has generated the hypothesis that the patient activation process, its antecedents, and its consequences on patient care, are disease-transcending instead of disease-specific. [28]

A great deal of effort is being devoted to implementing programs and initiatives intended to improve patients’ activation as a crucial predictor of medication adherence and better healthcare utilization. But they often prove not to be effective.[28] The reason for this ineffectiveness is often attributed to the lack of personalization of patient activation interventions.[29] A further reason may relate to the lack of agreement on the factors associated with patient activation and which may promote it.[30] The variance empirically explained regarding the level of activation in patients with chronic illness is still low,[31] so that it is difficult to design interventions effective in promoting self-management. In particular, the vast majority of studies exploring the variables that may impact on patient activation are primarily related to extrinsic factors related to the patient condition. In this domain, studies have demonstrated the association between patient activation and socio-cultural characteristics of the individual patient such as gender, age, level of education[28,32] and level of income [12,33]. Other studies have demonstrated the association between patients’ activation and their clinical condition: such as type and year of diagnosis [28], type of treatment regimen [34], level of health literacy[35–37], and psychiatric and cognitive condition of the patient[38]. However, the results are often partial and contrasting, which impedes final consensus on the factors, which predict the patient activation level.

Only few studies have sought to assess the role of psychological factors in influencing patient activation[24,35,39,40], although the level of patient activation may not be ascribable solely to external, contextual causes. In this regard, some studies have verified that patients’ health locus of control[41,42]–defined as the individual’s set of beliefs and motivation concerning self-determination of his/her health–is associated with patients’ level of activation.[13,40] Similarly, the perceived level of social support received from informal caregivers or the patient’s peers has been demonstrated to be a predictor of patient activation.[31] Other studies have argued that the emotional state of patients in a specific life moment may influence their ability to assume a proactive role in their care management [43]. These studies have shown that a higher level of patient activation is associated with lower depressive symptoms, although they have not demonstrated the causal relationship (and its direction) between these two variables (patient activation and depressive symptoms). Finally, some studies have explored the role of the perceived quality of the patient/doctor relationship in determining patients’ activation: in particular, a reference healthcare professional perceived as open to dialogue, emotionally supportive, and easily accessible has been demonstrated to be associated with a higher level of patient activation.[14,44]

This preliminary scientific evidence on the psychological factors associated with patient activation is promising. They suggest that more personalized interventions aimed at improving patients’ self-management and adherence can be devised. However, only few of the studies currently available in the scientific arena have explored the causal relationship between these different factors and patient activation.[39,45,46] A more systematic modeling of the psychosocial factors causing patient activation is therefore needed. Furthermore, the concept of patient activation often overlaps with the one of patient engagement. However, systematic reviews of the scientific literature demonstrated that the two concepts are diverse and linked to different phenomena. Basing on previous studies, patients’ engagement refers to the ability of patients to give sense and to adjust to their disease and their actual care condition. It may play an important mediating role in the activation process and in patient adherence to treatment. In previous studies, we have explored how the experience of patient engagement emotionally develops in the care journey as a consequence of a complex sense-making process related to patients’ health status and perceived role in the healthcare journey. We have described this process in its four evolving phases (blackout, arousal, adhesion, eudaimonic project) and termed it the Patient Health Engagement Model (PHE-model).[44,47–50] This model highlights how patients emotionally elaborate and give sense their illness status and patients’ identity, and it may be considered a crucial precursor of their ability and willingness to play a more active role in self-management. A previous study has highlighted how PHE-model plays a mediating role in the activation of type-2 diabetes patients.[44]

**Fig 1:**
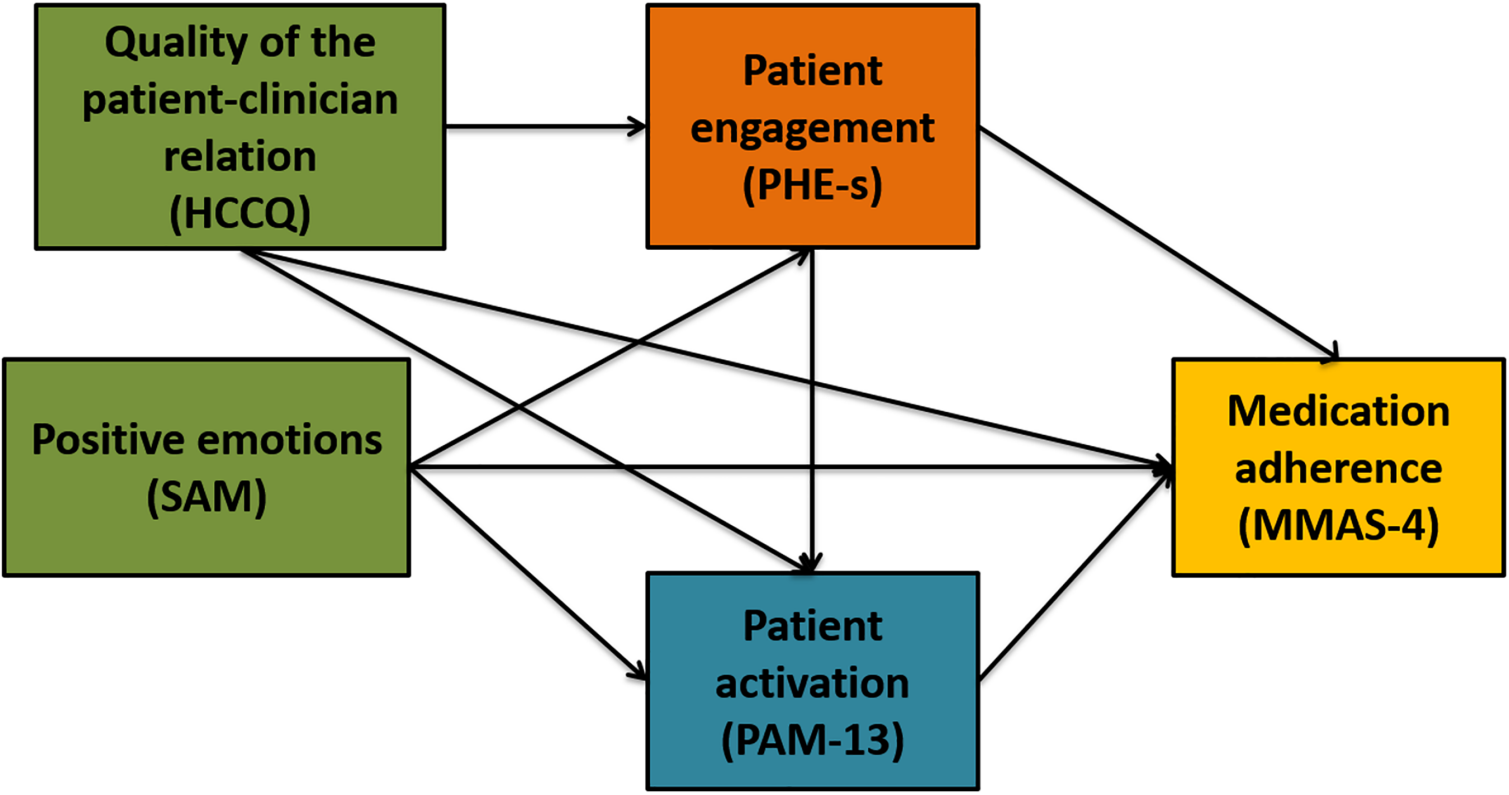
The a priori model tested in the current study. This is a path diagram describing the hypothesized effects of positive emotions and of the ability of the healthcare professionals to support patients’ autonomy on patient activation and medication adherence; it also describes the hypothesized mediating effect of patient engagement in this pathway. Unidirectional straight arrows indicate the predicted direction of the hypothesized effect. Note: HCCQ: *Health Care Climate Questionnaire*, PHE-s: *Patient Health Engagement Scale*, SAM: *Self-Assessment Manikin Scale*, PAM-13: *Patient Activation Measure-short form*, MMAS-4: *4 item-Morinsky Medication Adherence Scale*.

Based on these premises, we claim that the ability of the healthcare professional to legitimize the proactive role of patients in their healthcare might affect their reported adherence to medical treatments. This variable might also predict the level of patient activation. Furthermore, the quality of the emotional feelings experienced by patients in relation to their illness might impact on patient activation levels. Finally, we assume that the PHE-model—due to its ability to capture the psychological journey of patient engagement–may be a mediator of the impact of the quality of emotional feelings and of the perceived quality of the relationship with the healthcare professional on patient activation and on patient adherence (Fig 1).

To sum up, this study sought to verify the following theoretical hypotheses:

1. The level of patients’ activation affects patients’ adherence to treatment;
2. The quality of patients’ emotional feelings about the illness experience affects patient activation;
3. The level of perceived quality of the relationship with the reference healthcare professional affects patient activation and patient adherence;
4. The PHE-model mediates the impact of positive emotions and of perceived quality of the relationship with the healthcare professional on patient activation and on patient adherence.

## Methods

### Setting and participants

The research was conducted on a sample of 352 Italian-speaking chronic patients randomly selected from the Research Now Panel (http://www.researchnow.com/en-US.aspx). The entire Research Now Panel covers a wide range of chronic diseases and comprises more than 6.5 million registered subjects worldwide. The panel provider screens subjects belonging to the Research Now Panel for their authenticity via digital fingerprint and geo-IP-validation. All Research Now’s panelists are profiled on the basis of their socio-demographic, clinical and lifestyle characteristics. To ensure data reliability, the Research Now Panel is certified to be statistically representative of all the populations covered. For the specific purposes of this research, we randomly selected a sample of patients enrolled by the Research Now panel, according to the following pre-defined inclusion criteria: patients included in our study sample had to be Italian-speaking, affected by a chronic condition, aged over 18 years old, and of each gender. Patients with dementia, cognitive impairments, active psychiatric disorders, blindness, deafness, or insufficient Italian language skills to answer the questions meaningfully, or without informed consent, were excluded from this study. In order to be certain regarding our sample patients’ characteristics we asked them to confirm—before completing the study’s survey—previously collected by the Research Now Panel such as their demographics (i.e. gender, date and place of birth, ethnicity, nationality, educational level, place of residence) and clinical diagnosis.

The sample included in our study is not a stratified and fully representative of the Italian chronic population but it was randomly selected in order to guarantee its probabilistic feature. However, in this study we did not seek a descriptive estimation of the variables under examination, rather we were interested in their associative relationship. Thus, we did not consider the full representativeness of the sample necessarily required. All participants provided written informed consent before being enrolled in the research.

Initially, we randomly selected and enrolled a convenience sample of 500 patients according to the inclusion criteria established for this study. Only 352 returned the completed survey. Patients who did not complete the entire survey did not differ from the ones who provided all responses in terms of socio-demographic characteristics (Table 1).

**Table 1:** Summary of the sample characteristics.

Socio-demographic characteristics	Completers	Non completers
Age (years)	M = 53.1; DS = 15.1	55.2; DS = 16.3
Gender (% female)	46.1	54.7
Disease duration	M = 12.7; DS = 10.8	M = 14.2; DS = 9.7
**Marital Status (%)**		
Never married	21.2	22.6
Married	69.0	67.3
Divorced	7.8	7.4
Widowed	2.0	2.7
**Employment (%)**		
Employed	46.5	48.8
Retired	30.8	28.5
Housewife	8.4	7.9
Student	4.7	4.5
Unemployed	6.4	7.9
Other	3.2	2.4
**Education (%)**		
Elementary School or none	6.6	5.9
Junior High School	12.5	16.7
High School	48.1	42.8
College Education	29.0	31.5
PHD or Master	3.8	3.1
**Clinical variables** (Disease condition, %)		
Asthma	25.5	
Celiac disease	4.8	
Hypertension	35.6	
COPD	8.1	
Type I diabetes	3.7	
Type II diabetes	24.2	
Cardiovascular disorder	15.3	
Cancer	9.6	
Chron’s disease	2.9	
Fibromyalgia	7.6	
Ulcerous colitis	4.5	
Lupus	2.2	
Osteoarthritis	10.8	
Rheumatoid arthritis	11.1	
Myeloid chronic leukaemia	0.6	
Hypercholesterolemia	22.1	
Hepatitis	3.4	
Anaemia	9.3	
**Psychometric variables**		
PHE–S	Median = 3 (range 1–4); Entropy = .83; Ordinal Alpha = .84; Skewness = -.08; Kurtosis = -.44	
PAM-13	M = 65.3 (range 0–100); DS = 16.8; Cronbach’s Alpha = .89; Skewness = -.17; Kurtosis = .69	
MMAS-4	M = 1.3 (range 0–4); DS = 1.3; Ordinal Alpha = .78; Skewness = .71; Kurtosis = -.67	
HCCQ	M = 63.9 (range 13–91); DS = 15.5; Cronbach’s Alpha = .93; Skewness = -.61; Kurtosis = .44	
SAM	M = 2.9 (range 1–5); DS = .9; Cronbach’s Alpha = .77; Skewness = .60; Kurtosis = .25	

### Survey development and main measures

The survey was based on a structured self-administered online questionnaire (powered by the QUALTRICS online platform, https://www.qualtrics.com/). The Research Now Panel provider mailed the online link to the survey to the enrolled participants.

The questionnaire developed by the Authors of this study included validated measures and *ad hoc* items. Following, a detailed description of the included measures:

- Patient Health Engagement Scale (PHE-S)[51]. This is a measure of patient engagement grounded in rigorous conceptualization and appropriate psychometric methods. The scale consists of 5 ordinal items and was developed on the basis of the authors’ conceptual model of patient engagement (PHE-model), which features four positions along a continuum of engagement (i.e. blackout; arousal; adhesion; eudaimonic project). These engagement positions result from the conjoint cognitive (think), emotional (feel), and conative (act) involvement of individuals in their health management. This instrument is today the only one specifically dedicated to assess the degree of emotional elaboration and adjustment reached by the patient concerning his/her own health condition when engaging in health management. The specificity of this scale lays in the fact that it allows not only to assess the current patient’s attitude towards his/her health condition, but also to forecast the patient’s risk for disengagement in health management and thus to design preventive targeted intervention to optimize care pathways. According to the ordinal nature of this scale, the median score is considered the more robust and reliable index to calculate the final PHE-s scoring [52]. To obtain the PHE-s level, the median of the row PHE-s scores should be calculated. And with a simple conversion it is possible to transform the row PHE-s scores in the corresponding patient’s engagement position [50,53–55].
- Patient Activation Measure (PAM-13) developed by Hibbard and colleagues [56,57]. The 13-item Patient Activation Measure is an interval-level, unidimensional Guttman-like measure that contains items measuring self-assessed knowledge about chronic conditions, beliefs about illness and medical care, and self-efficacy for self-management. PAM-13-13 yields a scaled score ranging from 0 to 100 that assigns a patient to one of four incremental levels of patient activation (level 1: score of 47.0 or lower; level 2: score of 47.1 to 55.1; level 3: score of 55.2 to 67.0; level 4: score of 67.1 or above). The PAM-13 focuses on physical conditions, and it is designed to measure activation as a broad construct. In the present study, we used the Italian validated version of the PAM-13.
- Morisky Medication Adherence Scale (MMAS-4)[58,59]. Medication-taking behavior was assessed using the 4-item Morisky Medication Adherence Scale. This is a 4-item self-reported scale used to assess patients’ medication-taking behavior. TheMMAS-4addressed the essential reasons for non-adherence including forgetting, carelessness, and stopping the drug when feeling better or worse. Response categories were yes and no for each item with a dichotomous response. Scores obtained from the MMAS-4 ranged from 0 to 4. Scores of 0,1 to 2, and 3 to 4 were classified as high, medium, and low adherence, respectively. In our research, we used the Italian validated version of the MMAS-4.
- Health Care Climate Questionnaire (HCCQ)[60]. This scale assesses patients' perceptions of the ability of the healthcare professionals to support their autonomy (versus ‘controllingness’) and to motivate their initiative in care management. Autonomy support is associated with improved patient adherence, outcomes, and satisfaction, and is a critical element of patient-centered communication. Items in the scale include judgments about whether the physician provided patients with options about their health, conveyed confidence to them in their ability to make changes important for their health, and tried to understand their perspectives before suggesting medical or behavioral changes. Items also assess whether the patient felt understood by the physician, and whether the physician encouraged the patient to ask questions The HCCQ consists of 15 items on a seven-point Likert scale ranging from strongly disagree to strongly agree. The patient’s score consists in the average score of the items composing the scale. According to the analysis instructions, a patient HCCQ’s score is calculated by taking the average of the individual item scores to yield a mean score between 1 to 7, after reversing the single-reverse item. Higher average scores represent a higher level of perceived autonomy support [61, 62]. The scale was first developed and validated on the diabetic population by Williams and colleagues [60].
- Self-Assessment Manikin Scale (SAM)[62]. The Self-Assessment Manikin (SAM) was used to assess the patients’ quality of emotional feelings (positive or negative) [63]. This scale has the value to eliminate much of the bias associated with verbal measures of emotions and it is quick and simple to use. This is a wide used a 5-point pictographic scale to assess emotions associated with an event (i.e. the patient’s illness experience). Moreover SAM scale pictorial representation being a more human like figure may direct to further reliable decision on experienced emotion. SAM ranges from a smiling, happy figure to a frowning, unhappy one. The version of the SAM used in the present study that has five icons that define a 9-point scale. Mean score are calculated to determine the patient’s quality of emotional feelings. Subjects are asked to make a mark on the circle provided below each of the emotion figure by thinking about their illness experience.
- Demographic and clinical characteristics. A set of *ad hoc* items was included in the questionnaire in order to collect socio-demographic and clinical characteristics of the study sample. These data were also used as screening variables in order to select panel respondent according to our inclusion criteria. These items assessed the following patients’ characteristics: age (<60; > = 60); gender (male or female); education (elementary school, junior high school, high school, college education, PhD or master degree); occupational status (employed, retired, housewife, student, unemployed, other); marital status (never married, married, divorced, widowed); clinical variables (disease condition).

### Statistical analysis

Data analysis was conducted in five steps. In the first step descriptive analyses were conducted, with particular reference to socio-demographic characteristics of the sample.

In a second step of the analysis, the psychometric properties of the instruments were assessed in terms of reliability by using Cronbach’s Alpha for metric variables or Ordinal Alpha via Empirical Copula for ordinal variables [44]. A Cronbach’s or Ordinal Alpha via Empirical Copula, which was higher than 0.7 was considered acceptable. An evaluation of floor and ceiling effects for each items and a measure of skewness and kurtosis for the total scores of each scale were also performed in order to test Gaussian assumptions of next analyses.

In a third step of analysis, Gender, Education, Age, Employment and Marital Status Differences on outcome variables (*PAM-13* and *MMAS-4*) were investigated. For gender and age factors a t-test was conducted; for other factors a univariate Anova. In a fourth step of analysis, correlations between all the considered variables were calculated. Since every instrument produced a metric score, the linear correlation coefficient *r* was calculated and evaluated with a significance test.

In the last step, a Structural Equation Model with observed variables using ML estimation method was implemented [45] in order to evaluate the relationships between the considered variables and to explore the theoretical model hypothesized (see the hypotheses stated above). Structural equation analysis was conducted to test the hypothesized relationships among variables, which are based on a theoretical model previously developed (Fig 1). In the model we considered *HCCQ* and SAM as exogenous variables, and mediator (*PHE*-S) and dependent variables (*PAM-13*, *MMAS-4*) as endogenous ones. The Goodness-of-fit indexes were examined through Chi square test, RMSEA, CFI and SRMR. Models with acceptable fit presented non-significant Chi square value, RMSEA < .08 CFI > .90 and SRMR < .08 [46]. The normed fit index (NFI), relative fit index (RFI), incremental fit index (IFI) and Tucker-Lewis index (TLI) were used as incremental fit measures. An incremental fit measure value > 0.9 indicated a `good' fit for the model. Parsimonious normed-of-fit index (PNFI) and parsimonious comparative fit index (PCFI) were used as parsimonious fit measures. A value > 0.05 was considered reasonable for a good model fit. To improve the goodness-of-fit, modification indices were considered.

Analysis was conducted with IBM SPSS 24.0 and AMOS 23.0.

### Ethical concerns

The study received approval from the Università Cattolica del Sacro Cuore Ethics Committee. Patients consented to participate in the study, and they were allowed to withdraw from the study whenever they wanted. The data were collected anonymously and analyzed in aggregated form.

## Results

Overall, 500 patients were invited to participate in the study and completely answered the questionnaire for the analysis. 352 patients (159 female) completed the survey, mean age 53.1 (±15.1), and years with mean disease duration of almost 12 years. Table 1 lists the socio-demographic, clinical and psychometric characteristics of the sample. Mean, standard deviation (unless otherwise indicated) and a suitable reliability index (Cronbach Alpha or Ordinal Alpha via Empirical Copula) are reported for all the psychometric measures considered. All the psychometric measures presented a good or excellent reliability, with a Cronbach or Ordinal Alpha ranging from .77 to .93. No floor and ceiling effects were detected. Asymmetry and Kurtosis indices were in the acceptable limits [-1; + 1].

A t-test was performed to determine whether there were significant differences by gender and age (under or over 65 years) related to the outcome variables *PAM-13* and *MMAS-4*. An univariate Anova was performed to investigate differences related to the outcome variables for educational, employment and marital status. The results are reported in Table 2. Socio-demo characteristics did not impact on *PAM-13* scores, while age and employment status had a significant effect on *MMAS-4*. In particular, older and retired subjects presented a lower level of treatment adherence.

**Table 2:** PAM-13 and MMAS-4 scores for socio-demo factors.

Socio-demo factors	*PAM-13* mean score	*MMAS*-4 mean score	p-value *PAM-13*	p-value *MMAS*-4
**Gender**			0.208	0.815
Male	66.31	1.27		
Female	64.02	1.31		
**Age**			0.606	0.004**
Under 65 years	65.03	1.39		
Over 65 years	66.19	0.88		
**Marital Status**			0.445	0.054
Never married	63.92	1.49		
Married	66.01	1.18		
Divorced	62.86	1.65		
Widowed	65.26	1.29		
**Employment**			0.831	0.039*
Employed	66.07	1.36		
Retired	63.91	1.03		
Housewife	65.05	1.14		
Student	67.97	1.50		
Unemployed	62.62	2.00		
Other	67.08	1.45		
**Education**			0.093	0.979
Elementary School or none	64.83	1.67		
Junior High School	55.25	1.30		
High School	64.18	1.30		
College Education	65.17	1.27		
PHD or Master	67.57	1.34		

^*^p < .05

^**^p < .01

Table 3 reports linear correlation coefficients between the psychometric variables considered.

**Table 3:** Linear correlation coefficients between psychometric measures and frequency of mhealth/ehealth use.

	HCCQ	SAM	PHE-S	PAM-13	MMAS-4
**HCCQ**	-	-.07	.31**	.39**	-.23**
**SAM**		-	-.39**	-.32**	.11*
**PHE-S**			-	.38**	-.15**
**PAM-13**				-	-.18**
**MMAS-4**					-

*p < .05

**p < .01

*HCCQ* presented a significant correlation with all the measures except *SAM*: a positive correlation with *PHE-S* and *PAM-13* and a negative correlation with *MMAS-4* were detected.

*SAM* had a significant positive correlation with *MMAS-4*, and a negative correlation with *PHE-S* and *PAM-13*. *PHE-S* showed a significant direct correlation with *HCCQ* and *PAM-13*, and a negative correlation with SAM and with *MMAS-4*. *PAM-13* had a significant direct correlation with all the measures except *SAM* and *MMAS-4*.

Considering the hypotheses to be tested in the study and the correlations between the psychometric measures detected, a Structural Equation Model was implemented to verify associations and relationship between the variables.

**Fig 2:**
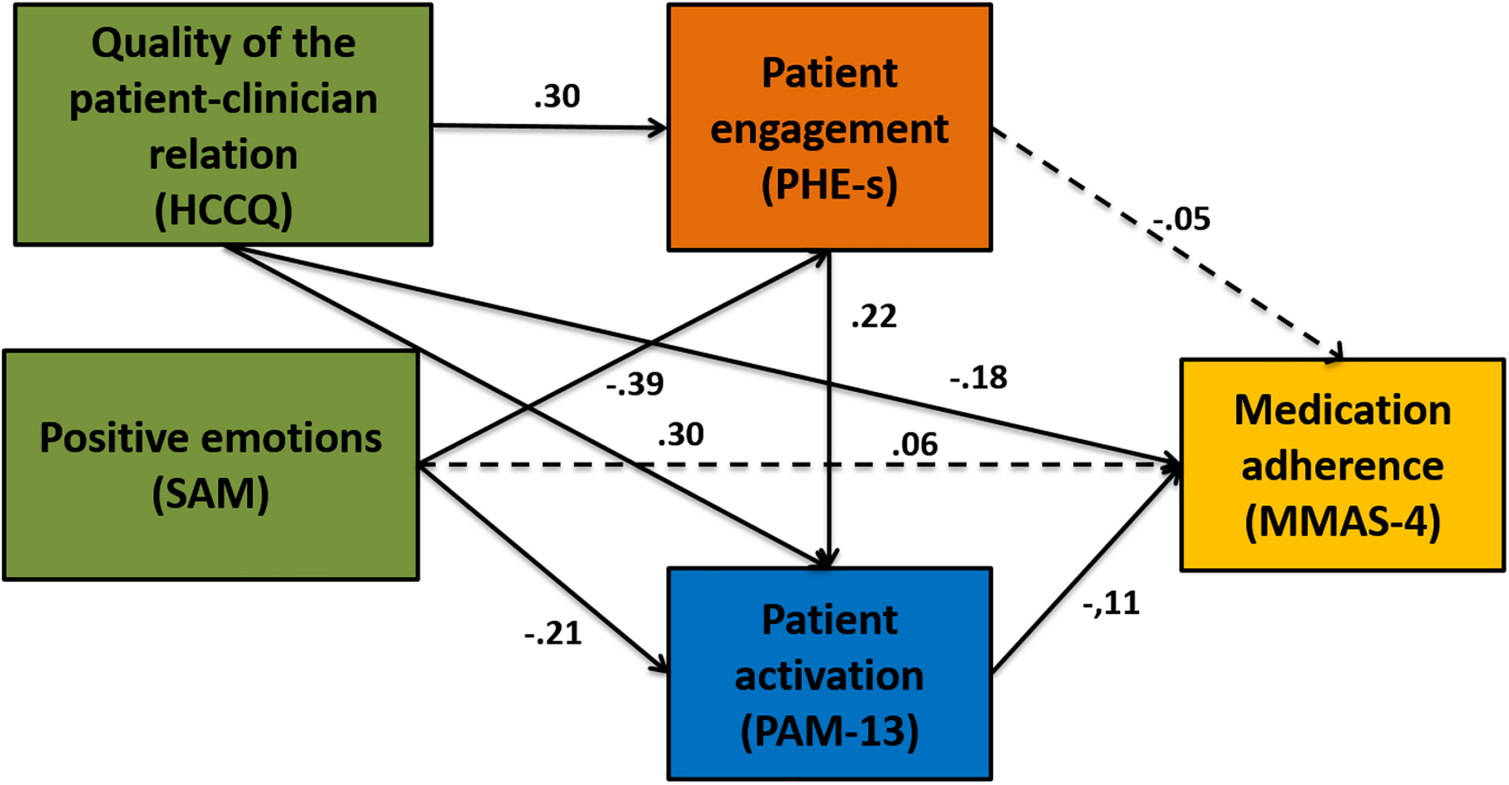
Significant pathways of the final model. Circles indicate unobserved latent variables, while rectangles represent observed variables. Significant paths with their estimated parameter are shown by solid lines. Standardized path coefficients are presented at the midpoint of the unidirectional arrow paths. Not significant paths are shown by dashed lines.

Relationships among patients' perceptions of the ability of the healthcare professionals to support their autonomy (*HCCQ*), negative patients’ emotions (*SAM*), patients’ engagement (*PHE-S*), patients’ activation (*PAM-13*) and medication adherence (*MMAS-4*) were tested. Fig 2 shows the explanatory model of the hypotheses that we wanted to verify.

The hypotheses were verified. Evaluation of the modification indexes did not suggest any change to the structure of the model.

The model presented an acceptable goodness-of-fit. Chi square test was not significant (χ^2^(1) = 1.9, *p* = .17). All goodness of fit indexes were satisfactory (RMSEA = 0.052, CFI = 0.996, SRMR = 0.040). The estimated paths were significant (*p* < .001). The Adjusted Goodness-of-fit (AGFI) was superior to .90 (AGFI = 0.967). NFI = 0.984, RFI = 0.940, IFI = 0.992, TLI = 0.960 as incremental fit measures, and PNFI = 0.099, PCFI = 0.100 as parsimonious fit measures confirmed the adequacy and consistency of the model. Some path coefficients have not been significant. Table 4 reports all standardized path coefficients, standard errors, 95% confidence intervals (via percentile bootstrap method) and p-value.

**Table 4:** Standardized path coefficients, standard errors, 95% confidence intervals (via percentile bootstrap method) and p-value.

Variable	*Standardized coefficients*	*Standard Error*	95% C.I.		p-value
			Lower bound	Upper bound	
**HCCQ→PHE-S**	.30	.05	.22	.37	< .01
**HCCQ→PAM**	.30	.06	.22	.38	< .01
**HCCQ→MMAS-4**	-.18	.06	-.28	-.08	< .01
**SAM→PHE-S**	-.39	.05	-.30	-.47	< .01
**SAM→PAM**	-.21	.05	-.30	-.12	< .01
**SAM→MMAS-4**	.06	.05	-.04	.13	.35
**PHE-S→PAM**	.22	.06	.13	.33	< .01
**PHE-S→MMAS-4**	-.05	.06	-.14	.06	.41
**PAM→MMAS-4**	.11	.05	-.20	-.02	.04

In addition to overall model fit, path coefficients also provide information regarding the direct and indirect effects. The direction of the arrow in Fig 2 implies the flow of the causal effect and the impact of one variable on another. Standardized path coefficients (i.e., the direct effect of a variable on the other) varied between-1 and +1 and could be interpreted in the same way as standardized multiple regression coefficients, indicating the amount of standard deviations a dependent variable will change, per standard deviation increase in the predictor Variable. Table 5 shows the standardized total, direct and indirect effects of each variable (estimates and standard errors) on the dependent variable *MMAS-4*.

**Table 5:** Standardized direct, indirect, and total effects of variables on the outcome variable *MMAS-4* (estimates and standard errors).

Variable	*Direct Effect*	*Indirect Effect*	Total Effect
**HCCQ**	-.18 (.06)	-.04 (.03)	-.22 (.05)
**SAM**	.06 (.05)	.04 (.03)	.10 (.05)
**PHE-S**	-.05 (.06)	-.03 (.02)	-.07 (.06)
**PAM-13**	-.11 (.06)	–	-.11 (.06)

## Discussion

The purpose of the analysis reported by this study was to contribute to the scholarly debate on the determinants of patient activation. In particular, given the crucial role that the level of patient activation plays in promoting the better quality and effectiveness of healthcare, the first objective was to verify the association between *PAM-13* level and patients’ reported adherence to treatment. Consistently with other previous studies [64,65], our results confirmed this relationship by demonstrating that patients’ activation is associated to their reported treatment adherence. This result is relevant to clinical practice because it confirms the importance of allocating time and effort to promoting patients’ empowerment and activation in order to assure their ability to self-manage and effectively adhere to treatment. In this study we verified the theoretical hypothesis and the hypnotized relationships among the variables included in our conceptual model. Structural equation analysis allows testing hypotesized relationships among variables, which are based on a theoretical model previously developed. Further research should be conducted in order to increase evidences about the direction of the hypotesized relationships among the variables. Indeed, reverse causality could be possible and should be tested by conducting longitudinal research design. For instance, further studies could be aimed at exploring the hypothesis that being empowered in their medical care cause patient to feel more positive in general and to experience higher quality patient-doctor relationships.

Furthermore, the analysis sought to disentangle the roles of psychosocial variables, such as positive emotion and perceived quality of the doctor/patient relationship, in determining the level of patients’ activation. We claimed that not only extrinsic factors such as patients’ demographic characteristics and their disease conditions might influence their level of activation.

In this framework, we primarily explored the role of positive emotional feelings in affecting patient activation, on the assumption that the psychological wellness of patients and their ability to adapt positively to their illness may be an antecedent of their ability to self-manage and assume an active role in the healthcare journey. This relationship was verified by confirming the role of the patients’ positive emotional state in affecting patient activation.

Furthermore, we explored the predictor role of the perceived quality of the patient/doctor relationship in sustaining patient activation. Also this relationship was verified, thus confirming the findings of previous studies [44,66–68]. In particular, the ability of healthcare professionals to motivate patients towards self-management and treatment adherence is important in this process; but especially so are their recognition and acceptance of the patients’ active role in the care journey. This study has demonstrated that the healthcare professional’s ability to make the patient autonomous in the care journey predicts the ability of patients to adopt a proactive role in the healthcare experience and to adhere to treatment [66,67,69]. This result suggests interesting further investigation on how the role of healthcare professionals and their attitudes to engagement are crucial assets with which to achieve activation.[70–75,68,67] Attitudes not given for granted since the concept of patient activation and engagement put into question the need of revisiting traditional power dynamics in the doctor-patients relationships [76,77].

A further purpose of our analysis was to explore the role of PHE-model in explaining patient activation. Particularly we proposed to consider Patient Health Engagement such as the patients’ psychological elaboration of their healthcare experience. In this study we explored the role of PHE-model in mediating the impact of positive emotions and the perceived quality of the doctor-patient relationship on the level of patient activation and medication adherence. Also this relationship was verified, confirming that PHE-model is a crucial factor in their ability to assume an active role in self-management and treatment adherence.

In previous studies, we argued that PHE-model must be conceived as a complex psychological process of adjustment to illness, which evolves in time and which is a function of several contextual factors [47]. In particular, engaging in the healthcare journey means becoming able not only to accept the diagnosis and its consequences for one’s health condition and lifestyle but also to understand one’s potential (starring) role in the care process. As a consequence, also the patient’s ability to become proactive in self-management and treatment adherence, thus improving his/her level of activation, is the result of a complex psychological elaboration and adjustment to the disease (and to the new”role patient”). Activation, therefore, may not be conceived as an ‘on/off’ state; it is determined by the developmental change in the patient’s identity on a complex journey of engagement. At the beginning of the care pathway, in fact, patients may be too overwhelmed and shocked (the ‘blackout’ phase in the PHE Model [50,78–80] to be able to assume an active role in the care process. In this phase patients tend to be passive and to delegate all decisions and actions concerning their care to the reference healthcare professionals. Activation or promotion of patient empowerment may be difficult and even counterproductive in this phase because the patient would reject medical attempts to make him or her autonomous.

With time, clinical support and education, the patient may then evolve in his/her adjustment to the disease and the care management. S/he moves to the state of psychological ‘alert’: here the patient is over-sensitized and worried about his/her ill body, and hyper-vigilant on signs and symptoms that may occur. In this phase, the patient is disorganized in his/her activation in a way likely to be dysfunctional for the clinical relationship and the care management. In this phase the patient is ‘disease centered’ because his/her psychological energy and cognitive resources are all focused on the disease, but s/he may be disorganized and over-demanding in his/her navigation of the healthcare system.

As their psychological adjustment evolves, patients acquire better mastery of their illness condition and improved awareness of their important role in determining the effectiveness of their care (‘adhesion’ phase). In this psychological phase, patients become ‘good patients’, with an acceptable literacy about their disease and its treatment and able to comply with the medical prescriptions. Although they are activated, they are not autonomous in self-management because they are still very reliant on their reference healthcare professional, whom they tend to over-consult upon even minimum changes in their everyday and care routines. Patients in this state tend to be still rather unsure about their role in self-determining the care journey and still reluctant to be autonomous in self-management.

A phase of full engagement then follows. Patients are completely aware of their health and care conditions, and also able to make satisfactory life plans despite the disease. According to the PHE-Model these patients are in a state of Eudaimonic Project. They have fully mastered their patient identity and agreed to play an active and fulfilling role in the care journey. But they have also become able to perceive themselves as persons, and not just as patients afflicted by a disease and by medical treatments. These patients have determined that ‘they are not their disease’, and this new psychological awareness foster energy, positive emotions and self-confidence in them.

Patients in this phase are fully aware that they are co-authors of their health. They accept that the effectiveness of care is also dependent on their motivation and determination to fight the disease and improve their quality of life. These fully engaged patients are well able to navigate the healthcare system and adhere to the medical prescription. They are also able to enact effective shared decision–making. [66,67,81,82]. Moreover, they become apostles of engagement practices, providing crucial testimony for other patients affected by the same disease but at the beginning of their engagement journey. These patients are fully activated and empowered towards their medical journey.

Concerning this study’s limitations, the heterogeneity of the diseases suffered by the patients in our sample may be regarded as a weakness. Furthermore, although the sample analyzed by our research was not stratified and fully representative of the Italian chronic population, it was randomly selected in order to guarantee its probabilistic nature. We used it only to explore the relationships among the variables under analysis (i.e. for associative purposes, not for descriptive estimation of their dimensions): given these considerations, full representativeness was not necessarily required. Moreover, in this study we explored formal mediation among the included variables. This is basically an observational study with no experimental manipulation of the independent variables involved in the conceptual model under investigation. Indeed, it is possible that several of the relationships between the variables included in the analysis are actually operating in a reverse manner from the hypothesized relationships (i.e., perhaps adherence increases patient activation through feeling the positive benefits of treatment) and the cross-sectional nature of the data preclude determining this. For these reasons, further longitudinal studies should be conducted to verify a causal relationship among the variables.

Furthermore, although the sample included in our study is not a stratified and fully representative of the Italian chronic population, it was randomly selected in order to guarantee its probabilistic feature. We used it only to explore the relationships of the variables under analysis (i.e. for associative purposes and not for a descriptive estimation of their dimensions): based on these considerations full representativeness is not necessarily required [83].

The PHE-model, measured in this study with the PHE-scale, casts light on possible psychological roots of patient motivation to self-management. The role the PHE-model in determining patient activation appears to us particularly promising for future research and clinical practice. PHE-model may be considered as lever to foster patients’ activation and—thus—patients’ adherence to treatments [84,85].

Our data suggest a cluster of factors associated with patient activation: mainly the level of patients’ elaboration of their disease, but also the quality of the patient/doctor relationship and positive emotional attitudes towards the health conditions [86]. If effective interventions to improve patient activation are to be developed, those targeting patients with lower levels of PHE-model should be prioritized [87].

## References

[1] Mosen DM, Schmittdiel J, Hibbard J, Sobel D, Remmers C, Bellows J. Is patient activation associated with outcomes of care for adults with chronic conditions? J Ambul Care Manage [Internet]. 2007;30(1):21–9. Available from: http://www.scopus.com/inward/record.url?eid=2-s2.0-33845773384&partnerID=tZOtx3y1 17170635 10.1097/00004479-200701000-00005

[2] Hibbard JH, Mahoney E. Toward a theory of patient and consumer activation. Patient Educ Couns [Internet]. 2010 3 [cited 2016 Jan 5];78(3):377–81. Available from: http://www.scopus.com/inward/record.url?eid=2-s2.0-77950350077&partnerID=tZOtx3y1 10.1016/j.pec.2009.12.015 20188505

[3] Hibbard JH. Engaging health care consumers to improve the quality of care. Med Care. 2003;41:I61–70. 12544817 10.1097/00005650-200301001-00007

[4] Greene J, Hibbard JH, Sacks R, Overton V, Parrotta CD. When patient activation levels change, health outcomes and costs change, too. Health Aff (Millwood) [Internet]. 2015 3 1 [cited 2015 Nov 16];34(3):431–7. Available from: http://www.scopus.com/inward/record.url?eid=2-s2.0-84924629092&partnerID=tZOtx3y1 25732493 10.1377/hlthaff.2014.0452

[5] Graffigna G, Barello S, Bonanomi A, Lozza E, Hibbard J. Measuring patient activation in Italy: Translation, adaptation and validation of the Italian version of the patient activation measure 13 (PAM13-I). BMC Med Inf Decis Mak [Internet]. 2015;15:109 Available from: http://www.ncbi.nlm.nih.gov/pmc/articles/PMC4690217/pdf/12911_2015_Article_232.pdf 10.1186/s12911-015-0232-9 PMC4690217 26699852

[6] Bandura A. Self-efficacy: Toward a unifying theory of behavioral change. Adv Behav Res Ther. 1978;1(4):139–61. 10.1037//0033-295x.84.2.191 847061

[7] Bandura A. Bandura Self-efficacy defined. In: Encyclopedia of Human Behavior [Internet]. 1994. p. 71–81. Available from: http://www.uky.edu/~eushe2/Bandura/BanEncy.html

[8] Rotter JB. Generalized expectancies for internal versus external control of reinforcement. Psychol Monogr Gen Appl. 1966;80(1):1–28. 5340840

[9] Rotter JB, Mulry RC. Internal versus external control of reinforcement and decision time. J Pers Soc Psychol. 1965;2(4):598–604. 5841368 10.1037/h0022473

[10] Prochaska JO, Velicer WF. The Transtheoretical Model of Health Behaviour Change. Vol. 12, American Journal of Health Promotion. 1997 p. 38–48. 10170434 10.4278/0890-1171-12.1.38

[11] Hibbard JH, Stockard J, Mahoney ER, Tusler M. Development of the Patient Activation Measure (PAM): conceptualizing and measuring activation in patients and consumers. Health Serv Res [Internet]. 2004;39(4 Pt 1):1005–26. Available from: http://www.pubmedcentral.nih.gov/articlerender.fcgi?artid=1361049&tool=pmcentrez&rendertype=abstract 15230939 10.1111/j.1475-6773.2004.00269.x PMC1361049

[12] Skolasky RL, Mackenzie EJ, Wegener ST, Riley LH. Patient activation and adherence to physical therapy in persons undergoing spine surgery. Spine (Phila Pa 1976) [Internet]. 2008 10 1 [cited 2016 Jan 3];33(21):E784–91. Available from: http://www.scopus.com/inward/record.url?eid=2-s2.0-65849299190&partnerID=tZOtx3y1 18827683 10.1097/BRS.0b013e31818027f1 PMC6153437

[13] Skolasky RL, Riley LH, Maggard AM, Bedi S, Wegener ST. Functional recovery in lumbar spine surgery: a controlled trial of health behavior change counseling to improve outcomes. Contemp Clin Trials [Internet]. 2013 9 [cited 2015 Nov 19];36(1):207–17. Available from: http://www.scopus.com/inward/record.url?eid=2-s2.0-84881005777&partnerID=tZOtx3y1 10.1016/j.cct.2013.06.018 23816487 PMC6153435

[14] Becker ER, Roblin DW. Translating primary care practice climate into patient activation: the role of patient trust in physician. Med Care [Internet]. 2008 8 [cited 2016 Jan 5];46(8):795–805. Available from: http://www.scopus.com/inward/record.url?eid=2-s2.0-49649111943&partnerID=tZOtx3y1 10.1097/MLR.0b013e31817919c0 18665059

[15] Pennarola BW, Rodday AM, Mayer DK, Ratichek SJ, Davies SM, Syrjala KL, et al Factors associated with parental activation in pediatric hematopoietic stem cell transplant. Med Care Res Rev [Internet]. 2012 4 [cited 2016 Jan 3];69(2):194–214. Available from: http://www.scopus.com/inward/record.url?eid=2-s2.0-84859549321&partnerID=tZOtx3y1 10.1177/1077558711431460 22203645 PMC4160822

[16] Remmers C, Hibbard J, Mosen DM, Wagenfield M, Hoye RE, Jones C. Is patient activation associated with future health outcomes and healthcare utilization among patients with diabetes? J Ambul Care Manage [Internet]. 2009;32(4):320–7. Available from: http://www.scopus.com/inward/record.url?eid=2-s2.0-74049091364&partnerID=tZOtx3y1 10.1097/JAC.0b013e3181ba6e77 19888008

[17] Harvey L, Fowles JB, Xi M, Terry P. When activation changes, what else changes? the relationship between change in patient activation measure (PAM) and employees’ health status and health behaviors. Patient Educ Couns [Internet]. 2012 8 [cited 2013 Sep 12];88(2):338–43. Available from: http://www.ncbi.nlm.nih.gov/pubmed/22459636 10.1016/j.pec.2012.02.005 22459636

[18] Hendriks M, Rademakers J. Relationships between patient activation, disease-specific knowledge and health outcomes among people with diabetes; a survey study. BMC Health Serv Res [Internet]. 2014 1 [cited 2016 Jan 3];14(1):393 Available from: http://www.scopus.com/inward/record.url?eid=2-s2.0-84908212353&partnerID=tZOtx3y1 25227734 10.1186/1472-6963-14-393 PMC4175625

[19] Shively MJ, Gardetto NJ, Kodiath MF, Kelly A, Smith TL, Stepnowsky C, et al Effect of patient activation on self-management in patients with heart failure. J Cardiovasc Nurs [Internet]. 2013 1 [cited 2016 Jan 3];28(1):20–34. Available from: http://www.scopus.com/inward/record.url?eid=2-s2.0-84871771789&partnerID=tZOtx3y1 10.1097/JCN.0b013e318239f9f9 22343209

[20] Smith SG, Curtis LM, Wardle J, von Wagner C, Wolf MS. Skill Set or Mind Set? Associations between Health Literacy, Patient Activation and Health. PLoS One. 2013;8(9). 10.1371/journal.pone.0074373 PMC3762784 24023942

[21] Maindal HT, Sandbæk A, Kirkevold M, Lauritzen T. Effect on motivation, perceived competence, and activation after participation in the Ready to Act” programme for people with screen-detected dysglycaemia: a 1-year randomised controlled trial, Addition-DK. Scand J Public Health [Internet]. 2011 5 [cited 2016 Jan 3];39(3):262–71. Available from: http://www.scopus.com/inward/record.url?eid=2-s2.0-79955137638&partnerID=tZOtx3y1 10.1177/1403494811402721 21427147

[22] Eikelenboom N, van Lieshout J, Wensing M, Smeele I, Jacobs AE. Implementation of personalized self-management support using the self-management screening questionnaire SeMaS; a study protocol for a cluster randomized trial. Trials [Internet]. 2013 1 [cited 2015 Dec 4];14(1):336 Available from: http://www.scopus.com/inward/record.url?eid=2-s2.0-84885534557&partnerID=tZOtx3y1 24134956 10.1186/1745-6215-14-336 PMC3874773

[23] Nijman J, Hendriks M, Brabers A, de Jong J, Rademakers J. Patient activation and health literacy as predictors of health information use in a general sample of Dutch health care consumers. J Health Commun [Internet]. 2014 1 [cited 2016 Jan 5];19(8):955–69. Available from: http://www.scopus.com/inward/record.url?eid=2-s2.0-84905913595&partnerID=tZOtx3y1 10.1080/10810730.2013.837561 24397280

[24] Skolasky RL, Mackenzie EJ, Wegener ST, Riley LH. Patient activation and functional recovery in persons undergoing spine surgery. J Bone Joint Surg Am [Internet]. 2011 9 21 [cited 2016 Jan 5];93(18):1665–71. Available from: http://www.scopus.com/inward/record.url?eid=2-s2.0-80053184825&partnerID=tZOtx3y1 10.2106/JBJS.J.00855 21938370

[25] Magnezi R, Glasser S. Psychometric properties of the hebrew translation of the patient activation measure (PAM-13). PLoS One. 2014;9(11). 10.1371/journal.pone.0113391 PMC4239053 25411841

[26] Solomon M, Wagner SL, Goes J. Effects of a Web-based intervention for adults with chronic conditions on patient activation: online randomized controlled trial. J Med Internet Res [Internet]. 2012 1 [cited 2016 Jan 3];14(1):e32 Available from: http://www.scopus.com/inward/record.url?eid=2-s2.0-84860554261&partnerID=tZOtx3y1 10.2196/jmir.1924 22353433 PMC3374536

[27] Hibbard JH, Greene J. What the evidence shows about patient activation: better health outcomes and care experiences; fewer data on costs. Health Aff (Millwood) [Internet]. 2013 2 [cited 2013 Sep 28];32(2):207–14. Available from: http://www.ncbi.nlm.nih.gov/pubmed/23381511 23381511 10.1377/hlthaff.2012.1061

[28] Bos-Touwen I, Schuurmans M, Monninkhof EM, Korpershoek Y, Spruit-Bentvelzen L, Ertugrul-van der Graaf I, et al Patient and disease characteristics associated with activation for self-management in patients with diabetes, chronic obstructive pulmonary disease, chronic heart failure and chronic renal disease: a cross-sectional survey study. PLoS One [Internet]. 2015 1 [cited 2015 Nov 11];10(5):e0126400 Available from: http://www.scopus.com/inward/record.url?eid=2-s2.0-84929169445&partnerID=tZOtx3y1 10.1371/journal.pone.0126400 25950517 PMC4423990

[29] Bos-Touwen I, Jonkman N, Westland H, Schuurmans M, Rutten F, de Wit N, et al Tailoring of Self-Management Interventions in Patients With Heart Failure. Vol. 12, Current Heart Failure Reports. 2015 p. 223–35. 10.1007/s11897-015-0259-3 25929690 PMC4424272

[30] Gary TL, Genkinger JM, Guallar E, Peyrot M, Brancati FL. Meta-Analysis of Randomized Educational and Behavioral Interventions in Type 2 Diabetes. Diabetes Educ [Internet]. 2003;29(3):488–501. Available from: http://tde.sagepub.com/cgi/doi/10.1177/014572170302900313 10.1177/014572170302900313 12854339

[31] Bos-Touwen I, Schuurmans M, Monninkhof EM, Korpershoek Y, Spruit-Bentvelzen L, Ertugrul-van der Graaf I, et al Patient and disease characteristics associated with activation for self-management in patients with diabetes, chronic obstructive pulmonary disease, chronic heart failure and chronic renal disease: a cross-sectional survey study. PLoS One [Internet]. 2015;10(5):e0126400 Available from: http://www.ncbi.nlm.nih.gov/pubmed/25950517 10.1371/journal.pone.0126400 25950517 PMC4423990

[32] Hibbard JH, Greene J, Tusler M. Plan design and active involvement of consumers in their own health and healthcare. Am J Manag Care [Internet]. 2008;14(11):729–36. Available from: http://www.scopus.com/inward/record.url?eid=2-s2.0-56749087989&partnerID=tZOtx3y1 18999907

[33] Rask KJ, Ziemer DC, Kohler SA, Hawley JN, Arinde FJ, Barnes CS. Patient activation is associated with healthy behaviors and ease in managing diabetes in an indigent population. Diabetes Educ [Internet]. 2009 1 [cited 2016 Jan 3];35(4):622–30. Available from: http://www.scopus.com/inward/record.url?eid=2-s2.0-69549110542&partnerID=tZOtx3y1 10.1177/0145721709335004 19419972

[34] Prey JE, Qian M, Restaino S, Hibbard J, Bakken S, Schnall R, et al Reliability and validity of the patient activation measure in hospitalized patients. Patient Educ Couns. 2016; 10.1016/j.pec.2016.06.029 PMC5121059 27422339

[35] Smith SG, Curtis LM, Wardle J, von Wagner C, Wolf MS. Skill Set or Mind Set? Associations between Health Literacy, Patient Activation and Health. PLoS One [Internet]. 2013 1 [cited 2013 Sep 24];8(9):e74373 Available from: http://www.pubmedcentral.nih.gov/articlerender.fcgi?artid=3762784&tool=pmcentrez&rendertype=abstract 10.1371/journal.pone.0074373 24023942 PMC3762784

[36] Alegría M, Sribney W, Perez D, Laderman M, Keefe K. The role of patient activation on patient-provider communication and quality of care for US and foreign born Latino patients. J Gen Intern Med [Internet]. 2009 11 [cited 2014 Nov 8];24 Suppl 3:534–41. Available from: http://www.pubmedcentral.nih.gov/articlerender.fcgi?artid=2764038&tool=pmcentrez&rendertype=abstract 19842003 10.1007/s11606-009-1074-x PMC2764038

[37] Prey JE, Woollen J, Wilcox L, Sackeim AD, Hripcsak G, Bakken S, et al Patient engagement in the inpatient setting: a systematic review. J Am Med Inform Assoc [Internet]. 2013;1–9. Available from: http://www.ncbi.nlm.nih.gov/pubmed/24272163 24272163 10.1136/amiajnl-2013-002141 PMC4078275

[38] McCusker J, Lambert SD, Cole MG, Ciampi A, Strumpf E, Freeman EE, et al Activation and Self-Efficacy in a Randomized Trial of a Depression Self-Care Intervention. Health Educ Behav [Internet]. 2016;1090198116637601-. Available from: http://heb.sagepub.com/content/early/2016/05/11/1090198116637601.full 10.1177/1090198116637601 27179288

[39] Goodworth M-CR, Stepleman L, Hibbard J, Johns L, Wright D, Hughes MD, et al Variables associated with patient activation in persons with multiple sclerosis. J Health Psychol [Internet]. 2016 1 [cited 2016 Jan 5];21(1):82–92. Available from: http://www.scopus.com/inward/record.url?eid=2-s2.0-84951066140&partnerID=tZOtx3y1 10.1177/1359105314522085 24591120

[40] Skolasky RL, Mackenzie EJ, Wegener ST, Riley 3rd LH. Patient activation and adherence to physical therapy in persons undergoing spine surgery. Spine (Phila Pa 1976) [Internet]. 2008;33(21):E784–91. Available from: http://www.ncbi.nlm.nih.gov/pubmed/18827683 18827683 10.1097/BRS.0b013e31818027f1 PMC6153437

[41] Smith PB, Trompenaars F, Dugan S. The Rotter locus of control scale in 43 countries: a test of cultural relativity. Int J Psychol [Internet]. 1995;30(3):377–400. Available from: 10.1080/00207599508246576

[42] Lefcourt HM. Locus of control. Measures of personality and social psychological attitudes. 1991. p. 413–99.

[43] Blakemore A, Hann M, Howells K, Panagioti M, Sidaway M, Reeves D, et al Patient activation in older people with long-term conditions and multimorbidity: correlates and change in a cohort study in the United Kingdom. BMC Health Serv Res [Internet]. 2016;16(1):582 Available from: http://bmchealthservres.biomedcentral.com/articles/10.1186/s12913-016-1843-2 10.1186/s12913-016-1843-2 27756341 PMC5069882

[44] Graffigna G, Barello S, Bonanomi A, Menichetti J. The Motivating Function of Healthcare Professional in eHealth and mHealth Interventions for Type 2 Diabetes Patients and the Mediating Role of Patient Engagement. J Diabetes Res. 2016; 2016 Available from: 10.1155/2016/2974521 PMC4736395 26881243

[45] Parchman ML, Palmer RF. Activation, Medication Adherence, and Intermediate Clinical Outcomes in Type 2. 2010;410–7. 10.1370/afm.1161 PMC2939416 20843882

[46] McCusker J, Lambert SD, Cole MG, Ciampi A, Strumpf E, Freeman EE, et al Activation and Self-Efficacy in a Randomized Trial of a Depression Self-Care Intervention. [Internet]. Health education & behavior: the official publication of the Society for Public Health Education. 2016 p. 1090198116637601-. Available from: http://heb.sagepub.com/content/early/2016/05/11/1090198116637601.full 10.1177/1090198116637601 27179288

[47] Graffigna G, Barello S, Triberti S. Patient engagement: A consumer-centered model to innovate healthcare. Patient Engagement: A Consumer-Centered Model to Innovate Healthcare. 2016. 1–141 p.

[48] Barello S, Graffigna G, Vegni E, Savarese M, Lombardi F, Bosio AC. “Engage me in taking care of my heart”: a grounded theory study on patient-cardiologist relationship in the hospital management of heart failure. BMJ Open [Internet]. 2015 1 [cited 2015 Nov 23];5(3):e005582 Available from: http://www.scopus.com/inward/record.url?eid=2-s2.0-84926461918&partnerID=tZOtx3y1 10.1136/bmjopen-2014-005582 25776041 PMC4369000

[49] Graffigna G, Barello S, Libreri C, Bosio CA. How to engage type-2 diabetic patients in their own health management: implications for clinical practice. BMC Public Health [Internet]. 2014 1 [cited 2016 Jan 5];14(1):648 Available from: http://www.scopus.com/inward/record.url?eid=2-s2.0-84902890694&partnerID=tZOtx3y1 24966036 10.1186/1471-2458-14-648 PMC4083034

[50] Barello S, Graffigna G. Engaging patients to recover life projectuality: an Italian cross-disease framework. 2015;24(5):1087–96 10.1007/s11136-014-0846-x 25373927

[51] Graffigna G, Barello S, Bonanomi A, Lozza E. Measuring patient engagement: development and psychometric properties of the Patient Health Engagement (PHE) Scale. Front Psychol [Internet]. 2015 1 [cited 2016 Jan 5];6(3):274 Available from: http://www.scopus.com/inward/record.url?eid=2-s2.0-84926457655&partnerID=tZOtx3y1 10.3389/fpsyg.2015.00274 25870566 PMC4376060

[52] Graffigna G, Barello S. The Value of Measuring Patient Engagement in Healthcare: New Frontiers for Healthcare Quality Promoting Patient Engagement and Participation for Effective Healthcare Reform In: Graffigna G, editor. Promoting Patient Engagement and Participation for Effective Healthcare Reform. Hershey PA, USA: IGI Global; 2016 p. 192–214.

[53] Graffigna G, Barello S, Libreri C, Bosio C a. How to engage type-2 diabetic patients in their own health management: implications for clinical practice. BMC Public Health [Internet]. 2014;14:648 Available from: http://www.ncbi.nlm.nih.gov/pubmed/24966036 10.1186/1471-2458-14-648 24966036 PMC4083034

[54] Barello S, Graffigna G, Vegni E, Savarese M, Lombardi F, Bosio a C. “Engage me in taking care of my heart”: a grounded theory study on patient-cardiologist relationship in the hospital management of heart failure. BMJ Open [Internet]. 2015;5(3):e005582 Available from: http://www.pubmedcentral.nih.gov/articlerender.fcgi?artid=4369000&tool=pmcentrez&rendertype=abstract 10.1136/bmjopen-2014-005582 25776041 PMC4369000

[55] Triberti S, Barello S. The quest for engaging AmI: Patient engagement and experience design tools to promote effective assisted living. J Biomed Inform [Internet]. 2016;63:150–6. Available from: 10.1016/j.jbi.2016.08.010 10.1016/j.jbi.2016.08.010 27515924

[56] Hibbard JH, Mahoney ER, Stockard J, Tusler M. Development and testing of a short form of the patient activation measure. Health Serv Res [Internet]. 2005 12 [cited 2015 Dec 12];40(6 Pt 1):1918–30. Available from: http://www.scopus.com/inward/record.url?eid=2-s2.0-29144521827&partnerID=tZOtx3y1 16336556 10.1111/j.1475-6773.2005.00438.x PMC1361231

[57] Hibbard JH, Stockard J, Mahoney ER, Tusler M. Development of the patient activation measure (PAM): Conceptualizing and measuring activation in patients and consumers. Health Serv Res [Internet]. 2004;39(4 I):1005–26. Available from: http://www.scopus.com/inward/record.url?eid=2-s2.0-3142668984&partnerID=tZOtx3y1 15230939 10.1111/j.1475-6773.2004.00269.x PMC1361049

[58] Tan X, Patel I, Chang J. Review of the four item Morisky Medication Adherence Scale (MMAS-4) and eight item Morisky Medication Adherence Scale (MMAS-8). Inov Pharm [Internet]. 2014;5(3). Available from: http://pubs.lib.umn.edu/innovations/vol5/iss3/5

[59] Castellucci LA, Shaw J, Van Der Salm K, Erkens P, Le Gal G, Petrcich W, et al Self-reported adherence to anticoagulation and its determinants using the Morisky medication adherence scale. Thromb Res. 2015;136(4):727–31. 10.1016/j.thromres.2015.07.007 26272305

[60] Williams GC, Freedman ZR, Deci EL. Supporting autonomy to motivate patients with diabetes for glucose control. Diabetes Care. 1998;21(10):1644–51. 9773724 10.2337/diacare.21.10.1644

[61] Schmidt K, Gensichen J, Petersen JJ, Szecsenyi J, Walther M, Williams G, et al Autonomy support in primary care—Validation of the German version of the Health Care Climate Questionnaire. J Clin Epidemiol. 2012;65(2):206–11. 10.1016/j.jclinepi.2011.06.003 21862287

[62] Bradley MM, Lang PJ. Measuring emotion: The self-assessment manikin and the semantic differential. J Behav Ther Exp Psychiatry. 1994;25(1):49–59. 7962581 10.1016/0005-7916(94)90063-9

[63] Tajadura-Jiménez A, Grehl S, Tsakiris M. The other in me: Interpersonal multisensory stimulation changes the mental representation of the self. PLoS One. 2012;7. 10.1371/journal.pone.0040682 PMC3404924 22866177

[64] Greene J, Hibbard JH. Why does patient activation matter? An examination of the relationships between patient activation and health-related outcomes. J Gen Intern Med. 2012;27(5):520–6. 10.1007/s11606-011-1931-2 22127797 PMC3326094

[65] Hibbard JH, Greene J, Shi Y, Mittler J, Scanlon D. Taking the long view: how well do patient activation scores predict outcomes four years later? Med Care Res Rev [Internet]. 2015 6 [cited 2016 Jan 3];72(3):324–37. Available from: http://www.scopus.com/inward/record.url?eid=2-s2.0-84930856664&partnerID=tZOtx3y1 10.1177/1077558715573871 25716663

[66] Barello S, Graffigna G, Pitacco G, Mislej M, Cortale M, Provenzi L. An Educational Intervention to Train Professional Nurses in Promoting Patient Engagement: A Pilot Feasibility Study. Front Psychol. 2017;7(1):2020 10.3389/fpsyg.2016.02020 28119644 PMC5222845

[67] Lamiani G, Barello S, Vegni E, Moja EA. “Diabetes is for me…”: The health care workers perspective | “Il diabete? per me…”: La prospettiva degli operatori sanitari. Assist Inferm e Ric. 2009;28(3). 20050500

[68] Barello S, Graffigna G, Vegni E, Savarese M, Lombardi F, Bosio AC. “Engage me in taking care of my heart”: a grounded theory study on patient–cardiologist relationship in the hospital management of heart failure. BMJ open. 2015 3 1;5(3):e005582 10.1136/bmjopen-2014-005582 25776041 PMC4369000

[69] Graffigna G, Barello S, Triberti S. Patient engagement: A consumer-centered model to innovate healthcare. Patient Engagement: A Consumer-Centered Model to Innovate Healthcare. 2016.

[70] Provenzi L, Barello S, Fumagalli M, Graffigna G, Sirgiovanni I, Savarese M, et al A Comparison of Maternal and Paternal Experiences of Becoming Parents of a Very Preterm Infant. JOGNN—J Obstet Gynecol Neonatal Nurs. 2016;45(4). 10.1016/j.jogn.2016.04.004 27266963

[71] Rademakers J, Jansen D, van der Hoek L, Heijmans M. Clinicians’ beliefs and attitudes toward patient self-management in the Netherlands; translation and testing of the American Clinician Support for Patient Activation Measure (CS-PAM). BMC Health Serv Res [Internet]. 2015 1 [cited 2016 Jan 5];15(1):138 Available from: http://www.scopus.com/inward/record.url?eid=2-s2.0-84928783944&partnerID=tZOtx3y1 25889832 10.1186/s12913-015-0799-y PMC4419501

[72] Stoilkova-Hartmann A, Janssen DJA, Franssen FME, Spruit MA, Wouters EFM. Attitudes of healthcare professionals providing pulmonary rehabilitation toward partnership in care. Heart Lung [Internet]. 2015 1 [cited 2016 Jan 3];44(4):347–52. Available from: http://www.scopus.com/inward/record.url?eid=2-s2.0-84930820615&partnerID=tZOtx3y1 10.1016/j.hrtlng.2015.05.003 26025762

[73] Hibbard JH, Collins PA, Mahoney E, Baker LH. The development and testing of a measure assessing clinician beliefs about patient self-management. Heal Expect. 2010;13(1):65–72. 10.1111/j.1369-7625.2009.00571.x PMC5060511 19906211

[74] Lamiani G, Barello S, Browning DM, Vegni E, Meyer EC. Uncovering and validating clinicians’ experiential knowledge when facing difficult conversations: A cross-cultural perspective. Patient Educ Couns. 2012;87(3):307–12. 10.1016/j.pec.2011.11.012 22196987

[75] Barello S, Graffigna G. Engagement-sensitive decision making: Training doctors to sustain patient engagement in medical consultations. Patient Engagement: A Consumer-Centered Model to Innovate Healthcare. 2016.

[76] Sorrentino M, Guglielmetti C, Gilardi S, Marsilio M. Health Care Services and the Coproduction Puzzle: Filling in the Blanks. Adm Soc [Internet]. 2015;0095399715593317-. Available from: http://aas.sagepub.com/cgi/content/long/0095399715593317v1

[77] Batalden M, Batalden P, Margolis P, Seid M, Armstrong G, Opipari-Arrigan L, et al Coproduction of healthcare service. BMJ Qual Saf [Internet]. 2016;25(7):509–17. Available from: http://www.ncbi.nlm.nih.gov/pubmed/26376674 10.1136/bmjqs-2015-004315 26376674 PMC4941163

[78] Graffigna G, Barello S. Innovating healthcare in the era of patient engagement: Challenges, opportunities & new trends. Patient Engagement: A Consumer-Centered Model to Innovate Healthcare. 2016.

[79] Graffigna G, Barello S, Riva G, Bosio AC. Patient Engagement: The Key to Redesign the Exchange between the Demand and Supply for Healthcare in the Era of Active Ageing. Vol. 203, Studies in Health Technology and Informatics. 2014. 26630515

[80] Graffigna G, Barello S, Triberti S. Giving (back) a role to patients in the delivery of healthcare services: Theoretical roots of patient engagement. Patient Engagement: A Consumer-Centered Model to Innovate Healthcare. 2016.

[81] Graffigna G., Barello S., Bonanomi A., & Riva G. (2017). Factors affecting patients’ online health information-seeking behaviors: the role of the Patient Health Engagement (PHE) Model. Patient Education and Counseling. 10.1016/j.pec.2017.05.033 (*in press*) 28583722

[82] Guyatt GH, Mulla SM, Scott IA, Jackevicius CA, You JJ. Patient engagement and shared decision-making. Vol. 29, Journal of General Internal Medicine. 2014 p. 562 10.1007/s11606-013-2727-3 24464283 PMC3965760

[83] Lozza E, Libreri C, Bosio CA. Temporary employment, job insecurity and their extraorganizational outcomes. Econ Ind Democr. 2013;34(2):89–105.

[84] Barello S, Graffigna G, Savarese M, Bosio AC. Engaging patients in health management: towards a preliminary theoretical conceptualization. Psicologia della Salute; 2015; 3:11–33.

[85] Barello S, Triberti S, Graffigna G, Libreri C, Serino S, Hibbard J, et al eHealth for patient engagement: A Systematic Review. Vol. 6, Frontiers in Psychology. 2016. 10.3389/fpsyg.2015.02013 PMC4705444 26779108

[86] Graffigna G, Barello S, Riva G, Savarese M, Menichetti J, Castelnuovo G, et al, Front. Psychol. 2017; 8:812 10.3389/fpsyg.2017.00812 28634455 PMC5460315

[87] Menichetti J, Graffigna G. “PHE in Action”: Development and Modeling of an Intervention to Improve Patient Engagement among Older Adults. Front Psychol [Internet]. 2016;7(9):1405 Available from: http://journal.frontiersin.org/article/10.3389/fpsyg.2016.01405 10.3389/fpsyg.2016.01405 27695435 PMC5025533

